# Structural determination of lipid-bound factor VIII

**DOI:** 10.1016/j.jtha.2026.05.021

**Published:** 2026-05-26

**Authors:** Bassem M. Mohammed

**Affiliations:** Edward A. Doisy Department of Biochemistry and Molecular Biology, Saint Louis University School of Medicine, St Louis, Missouri, USA

**Keywords:** cryoelectron microscopy, factor VIII, hemophilia A, membranes, phospholipids

## Abstract

**Background:**

Coagulation factor (F)VIII is the inactive precursor of FVIIIa, an essential component of the intrinsic tenase complex. Despite numerous structures of recombinant FVIII in solution, the structural basis of the procofactor–membrane interface remains poorly defined. Traditional liposome platforms require special grids and often suffer from heterogeneous particle distribution and overcrowding, precluding high-resolution single-particle analysis, while membrane mimetics—nanodiscs—are too planar, posing significant biophysical challenges.

**Objectives:**

This study established a reproducible methodology for structural determination of membrane-bound coagulation factors and resolve the procofactor FVIII–lipid interface.

**Methods:**

We used methylated branched lipids to compensate for membrane planarity and used cryoelectron microscopy to solve the structure of FVIII bound to lipids.

**Results:**

We resolved the cryoelectron microscopy structure of procofactor FVIII on nanodiscs and liposomes lipid membranes at 3.46 and 3.56 Å, respectively. Structural analysis reveals a definitive tilted docking orientation where both C domains mediate shallow interfacial insertion, with the C2 domain acting as the primary anchor and the C1 domain serving as a secondary tether. This spatial arrangement structurally explains the high clinical severity of C2 interface mutations compared with more moderate C1 surface mutations.

**Conclusion:**

Methylated branched lipids enable rapid, high-resolution characterization of membrane-associated clotting factors. This structure provides the first definitive template for the FVIII–lipid interface, serving as a benchmark for future studies on tenase complex assembly.

## 1 | INTRODUCTION

Factor (F)VIII is a large, multidomain glycoprotein featuring an A1-A2-B-A3-C1-C2 domain organization and serves as the essential procofactor in the intrinsic pathway of blood coagulation [1,2]. Upon activation to FVIIIa, it assembles with activated FIX (FIXa) to form the intrinsic tenase complex on the surface of procoagulant membranes. This assembly is a critical regulatory step, accelerating the activation of FXa by several orders of magnitude [3,4]. Congenital deficiency of FVIII result in hemophilia A, a severe bleeding disorder, traditionally managed with recombinant replacement therapies.

While several structures of FVIII have been characterized [5–9], the structural basis of the procofactor–membrane interface remains a significant blind spot in the field presented with unique biophysical challenges. Traditional liposome-based cryoelectron microscopy (cryo-EM) is often hindered by heterogeneous particle distribution, where few vesicles sequester the majority of the protein. Additionally, liposome (LP) samples typically require tedious grid optimization and/or the use of gold grids, which are frequently in short supply. Conversely, small membrane mimetics, nanodiscs (NDs), offer a more stable alternative for single-particle analysis but are planar in nature and often fail to replicate the high-curvature environment and lipid-packing defects required for coagulation protein docking. Linear lipids traditionally used (natural or synthetic) pack too tightly in planar geometries, effectively closing the hydrophobic gaps required for the insertion of the FVIII C–domain anchors/spikes [10–12].

In this study, we describe a rapid and reproducible methodology to overcome these constraints by usign methylated branched lipids. These lipids disrupt the tight acyl-chain packing, creating a curvature-mimetic environment on stable ND surfaces, compensating for the inherent planarity of the system. Using this pipeline, we resolved the cryo-EM structure of the FVIII on NDs at 3.46 Å resolution and on LPs at 3.56 Å. Our results reveal a definitive tilted docking orientation where the C2 domain acts as the primary anchor while the C1 domain serves as a secondary tether. This spatial arrangement structurally explains the high clinical severity of C2 interface mutations compared to more moderate C1 surface mutations.

## 2 | METHODS

### 2.1 | Sample preparation

Full-length recombinant human FVIII (Kogenate FS; Bayer HealthCare) was buffer exchanged into 20 mM HEPES pH 7.4, 150 mM NaCl, 5 mM CaCl_2_ by size exclusion chromatography using a Superdex 200 increase 10/300 GL column. Methylated lipids 1,2-diphytanoyl-sn-glycero-3-phosphocholine (DPhPC; Cat 850356) and 1,2-diphytanoyl-sn-glycero-3-phospho-L-serine (DPhPS; Cat 850408) were from Avanti Polar Lipids. NDs were reconstituted using membrane scaffold protein 1D1 (Cat M6574; Millipore sigma), with a DPhPC:DPhPS molar ratio of 80:20, following established protocols [13,14]. To ensure complete MSP saturation and stable disc formation, a slight molar excess of lipid is typically used. This often results in a heterogeneous ND preparations containing both lipid-saturated NDs and a subpopulation of residual LPs of approximately equal size. This heterogeneity enabled the internal validation of FVIII docking across 2 distinct membrane geometries: the constrained, planar ND surface and the relaxed, large-radius curvature of LPs, under identical conditions.

### 2.2 | Cryo-EM workflow

For EM studies, 3 μL of FVIII (0.1 mg/mL final concentration) mixed with 4 μM NDs were applied on Quantifoil R 1.2/1.3, Cu 300 mesh grids (EMS), which have been glow discharged for 20 seconds using atmospheric air and then plunge frozen in liquid ethane on a Vitrobot Mark IV (Thermo Fisher Scientific). Data collection was performed on Glacios 200 keV cryo-TEM. Single-particle analysis was performed in cryoSPARC v5.0.1 [15]. Following motion correction and contrast transfer function estimation, particles were subjected to extensive 2D and 3D classification to separate FVIII-ND and FVIII-LP populations. Final reconstructions reached global resolutions of 3.46 and 3.56 Å, respectively. Initial models were generated using FVIII Protein Data Bank (PDB) ID 3CDZ [5] and AlphaFold2 (AF-P00451). Individual domains were docked and rigid-body fitted in UCSF ChimeraXv1.10. For the 3.46 Å ND structure, all-atom refinement and manual model building were performed in Coot [16] using the composite map. For the 3.56 Å LP structure, a backbone trace was generated to reflect the global architecture. Both models underwent final real-space refinement in Phenix [17] using the AlphaFold model as a reference for secondary structure restraints. Data collection parameters, structure, and map statistics are summarized in Table.

### 2.3 | Data deposition

Atomic coordinates and cryo-EM maps have been deposited in the PDB and Electron Microscopy Data Bank (EMDB) under the following accession codes:

- Factor VIII–ND: PDB 12JP and EMD-76487 (composite map), EMD-76479 (consensus map), EMD-76482 (A1 domain focused map), EMD-76483 (A2 domain focused map), EMD-76484 (A3 domain focused map), EMD-76485 (C1 domain focused map), and EMD-76486 (C2 domain focused map).
- Factor VIII-LP: PDB ID 12JV and EMD-76497 (composite map), EMD-76495 (consensus map), EMD-76489 (A1 domain focused map), EMD-76490 (A2 domain focused map), EMD-76491 (A3 domain focused map), EMD-76492 (C1 domain focused map), and EMD-76494 (C2 domain focused map).

## 3 | RESULTS AND DISCUSSION

We resolved the cryo-EM structures of full-length FVIII on methylated branched-phospholipid NDs and LPs at resolutions of 3.46 and 3.56 Å, respectively (Figure 1A–C). Standard unsaturated phosphocholine and phospho-L-serine lipids of the dioleoyl or palmitoyl-oleoyl possess native *cis*-double bond kinks that impart a roughly conical molecular geometry; they, nonetheless, maintain a rather high lateral packing density in planar geometries. The tight acyl-chain alignment can preclude the high-resolution capture of membrane-binding interfaces by restricting the formation of hydrophobic gaps necessary for protein insertion [12,18,19]. To bypass these constraints, we tested methylated branched lipids, specifically DPhPC and DPhPS. The 4 methyl groups on the phytanoyl chains provide a permanent steric hindrance that disrupts regular lipid packing, a phenomenon characterized by a significantly increased population of persistent lipid-packing defects and enhanced membrane fluidity [20]. This disruption creates a curvature-mimetic environment on the surface of stable NDs, effectively replicating the hydrophobic accessibility of curved vesicles (Figure 1D) [21]. Applying this biophysical framework enabled the shallow insertion of FVIII C-domain anchors and the first high-resolution capture of the full-length procofactor in its membrane-bound state. The high-quality density maps for the ND-bound FVIII enabled unambiguous docking of individual domains and high-fidelity refinement of most side-chain orientations at the membrane interface as some were hindered by lipid membrane interactions. In contrast, while the LP-bound map clearly defined the global architecture, relatively lower local resolution necessitated a backbone trace for the majority of the model (Figure 2A, B). The defining feature of these structures is a definitive tilted orientation of the FVIII C-domains relative to the membrane. Structural alignment of these 2 models yielded a pruned Cα root mean square deviation of 0.935 Å (across 1113 atom pairs), confirming that the global observed tilted docking geometry across 2 distinct platforms is an intrinsic feature of the FVIII–membrane interaction (Figure 2C). Furthermore, the use of full-length FVIII provides the first high-resolution evidence that the B domain, although largely disordered and excluded from the final map, does not sterically interfere with the conserved C-domain docking mechanism, consistent with established functional data [1,2]. Despite a stable global architecture, we observed localized plasticity at the membrane interface encompassing residues 2200 to 2208 (the 2202 loop), which exhibited marked conformational divergence between the ND and LP structures (Figure 2C). While still maintaining membrane interactions, the 2202 loop exhibited a Cα displacement of up to 8.0Å at its apex (Pro2205). Understanding that our models represent the dominant conformers resolved in their respective maps, local densities suggests that these states likely coexist as part of a conformational ensemble across both membrane platforms with the 2202 loop of the C2 domain retaining a high degree of conformational freedom. This could be of biological significance, potentially, allowing the procofactor to adjust its position in response to varying degrees of membrane curvature or fluidity during the transition from an inactive procofactor to an active component of the tenase complex. Case in point, structural analysis of the hemophilia causing missense mutation A2201P suggests the proline residue could result in an early turn forcing a steric clash between the loop’s hydrophobic residues and the polar membrane interface. The mild bleeding phenotype observed is consistent with plasticity of the 2202 region that allowed toleration of this structural misalignment. This plasticity supports the design of FVIII variants with hobbled activity that was proposed as a strategy to avert immune responses while maintaining partial procoagulant function [22,23].

Our work builds directly upon the foundational membrane-bound models from Stoilova-McPhie laboratory that accurately identified the primary hydrophobic anchors at low resolution (15–20 Å) [24,25]. The structures in this study validate those findings while revealing a definitive tilted docking orientation at near atomic resolution. This tilt is likely a mechanical function of the asymmetric insertion pattern into the membrane rather than a large-scale domain rearrangement of the FVIII molecule itself. Indeed, the gross protein structure remains largely invariant compared with crystal structures, aside from a minor 2 to 3 Å shift along the *z*-axis of the C2 domain. The structures reveal the FVIII-C2 domain as the primary membrane anchor with 2 prominent hydrophobic spikes: Met2199/Phe2200 and Leu2251/Leu2252, mediating a shallow interfacial insertion (3–4 Å) toward the hydrophobic core of the membrane. The C1 domain acts as a secondary tether with a single spike: Lys2092/Phe2093. These insertions position the loops His2054-Ile2059 and Asp2131-Ser2132 of C1 and Gln2213-Ser2216, Gln2222-Asn2224, Asp2288-Phe2290, and Val2314-Gln2316 of C2 into a tangential contact with the lipid surface and facilitate membrane contacts with the basic residues Arg2090 and Lys2092 of C1 and Lys2207, Arg2215, Arg2220, and Lys2249 of C2. Notably, while the conserved Arg2163 (C1) and Arg2320 (C2) were previously proposed to facilitate direct membrane interaction [26], our steady-state reconstructions suggest these residues to play a role in peripheral loop stabilization rather than direct membrane binding. Alternatively, they may drive the initial electrostatic steering during the docking process. Structurally, the spatial differentiation between the C1 and C2 interfaces provides a direct explanation for the clinical heterogeneity seen in hemophilia A mutations [27]. Mutations located within the C2 spikes specifically residues Met2199, Phe2200, Leu2251, and Leu2252 are almost universally associated with severe phenotypes because they directly disrupt the primary anchor that dictates the entire molecule’s orientation. Conversely, mutations at the more tangential C1–membrane interface involving residue Lys2092 or within the more plastic 2202 loop of C2 often result in more moderate phenotypes.

The asymmetric arrangement is likely critical for the subsequent assembly of the intrinsic tenase complex, as it optimally orients the A domains to receive FIXa and then FX analogous to its homolog cofactor FVa in the prothrombinase complex (Figure 3A). Indeed, the tilted orientation observed in our FVIII structures is conserved with the homolog FVa. Alignment of our lipid-bound FVIII model with the membrane-binding C domains of FVa from the only reported membrane-bound prothrombinase complex (PDB 9CTH) [28] reveals a striking spatial overlap. The new structures dock perfectly into the prothrombinase map EMD-42405 (Figure 3B, C). This structural convergence suggests that this hierarchical, asymmetric docking is a fundamental functional requirement for the cofactor function to enhance the enzymatic efficiency of both the prothrombinase and intrinsic tenase complexes. By anchoring the procofactor via shallow insertion across both domains, the C domains create a conserved asymmetric cradle that optimally positions the catalytic domains of its subcomponents relative to their incoming substrates. These results provide a robust structural basis for modeling the complete intrinsic tenase complex while high-resolution cryo-EM data for the assembled complex are forthcoming.

The success of our branched-lipid approach directly addresses a persistent technical hurdle in the structural biology of coagulation complexes. Our previous work on the prothrombinase complex [28] used linear-lipid NDs and was significantly hindered by low particle counts. In hindsight, we hypothesize that using standard lipids likely rely on a subpopulation of nonideal discs, where stochastic packing defects or incomplete assembly inadvertently provide the hydrophobic access necessary for docking. The reliance on such stochastic sampling is prohibitively inefficient for routine high-resolution studies.

In summary, we have provided the first side-chain level visualization of the procofactor FVIII in its membrane-bound state. The structures reveal a definitive docking orientation where both C domains act as anchors through shallow penetration of the membrane. The remarkable structural convergence between our ND and LP models, alongside the spatial overlap with the prothrombinase complex (PDB 9CTH), establishes this asymmetric cradle as a fundamental motif in FV/FVa and FVIII/FVIIIa based complexes assembly. Ultimately, these findings not only resolve long-standing questions regarding FVIII docking but also provide a validated structural framework for modeling the assembled intrinsic tenase complex.

## Figures and Tables

**FIGURE 1:**
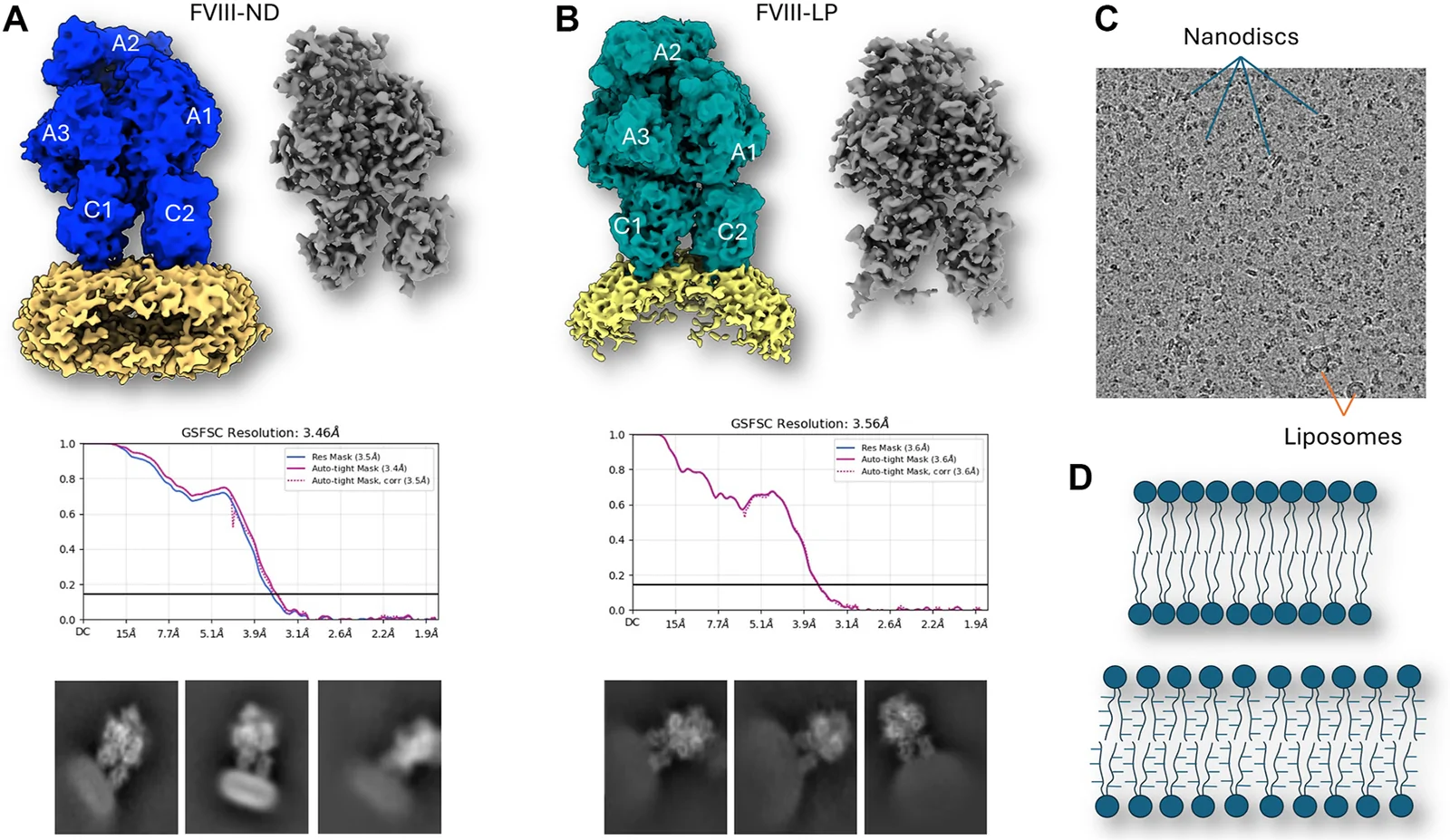
Cryoelectron microscopy (cryo-EM) structural determination of factor (F)VIII on nanodiscs and liposomes. (A) Consensus cryo-EM reconstruction of FVIII on nanodisc (EMD-76479, top left panel) and composite sharpened map (EMD-76487, top right panel). Middle panel: Gold-standard Fourier Shell correlation (GSFSC) for consensus map indicating a global resolution of 3.46 Å. Bottom panel: Representative reference-free 2D class averages showing clear secondary structural features of FVIII docked onto the nanodisc surface. (B) Same as (A) for FVIII on liposomes consensus map (EMD-76495) and composite sharpened map (EMD-76497). For both (A) and (B), the consensus map is shown at low isosurface to show the full lipid-membrane features. Factor VIII is annotated by its constituent domains. (C) Representative cryo-EM micrograph showing the coexistence of lipid-saturated nanodiscs and residual liposomes, enabling internal validation of FVIII docking across distinct membrane geometries. (D) Representative cartoon of a lipid bilayer showing tight acyl-chain packing exhibited by dioleoyl or palmitoyl-oleoyl lipids that restricts the formation of hydrophobic gaps (top panel) versus branched methylated lipids that forms curvature-mimetic defects necessary for the shallow interfacial insertion of FVIII C–domain spikes (bottom panel).

**FIGURE 2:**
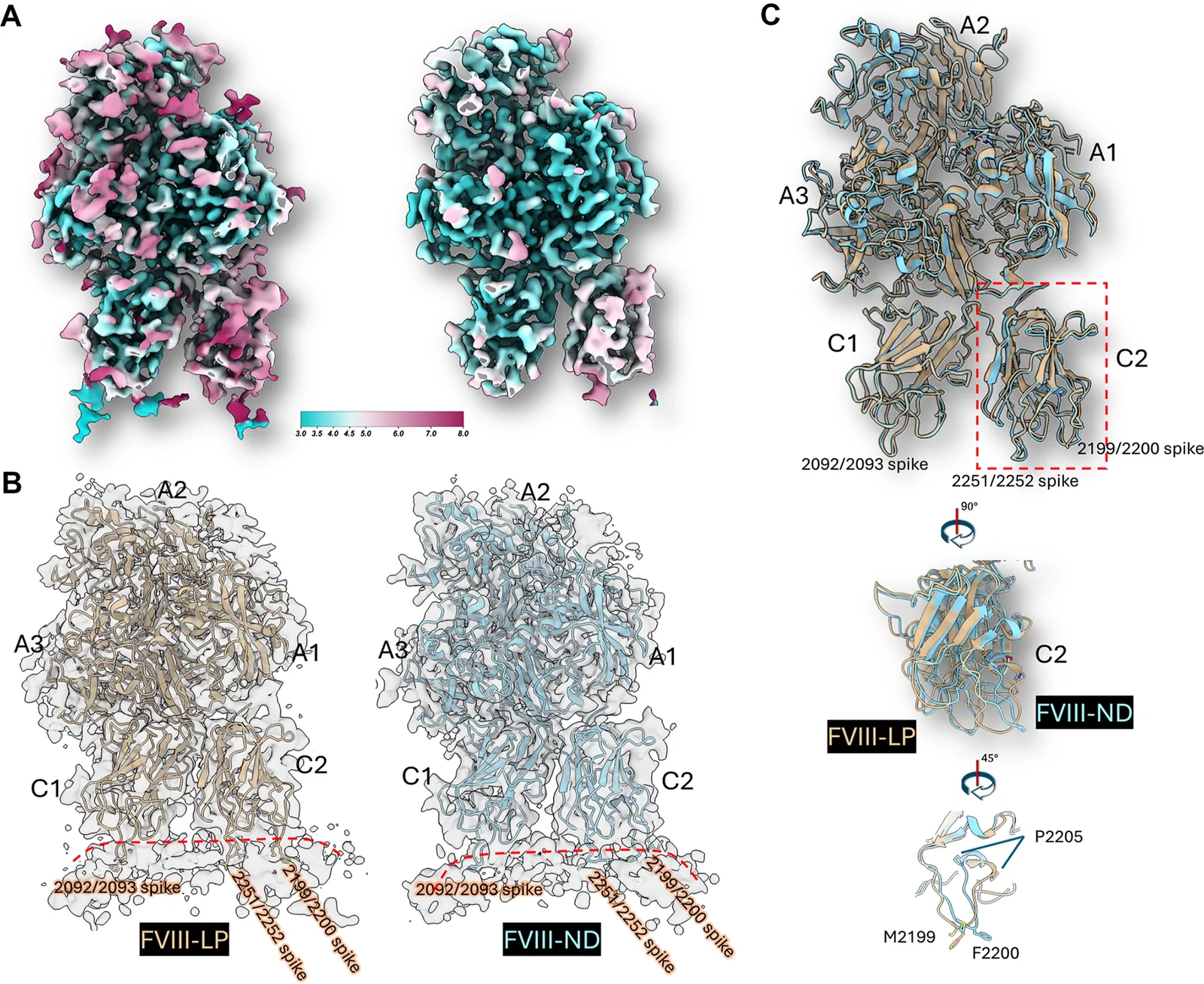
Factor (F)VIII model fitting and observed C2 plasticity. (A) Local resolution mapped onto the surface representation of EMD-76497 (FVIII–liposome [LP], left) and EMD-76487 (FVIII–nanodisc [ND], right). (B) Sharpened composite map (EMD-76497) for FVIII on liposomes with fitted PDB 12JV (left panel) and FVIII on nanodiscs using PDB 12JP composite map EMD-76487 (right panel), documenting the quality of the final model. A red dotted line marks the lipid–water interface, illustrating the shallow interfacial insertion (3–4 Å) of the hydrophobic spikes. The primary anchors, including spikes Lys2092/Phe2093 (C1) and Met2199/Phe2200 and Leu2251/Leu2252 (C2), are highlighted with residues shown in stick representation. (C) Overlay of the FVIII-ND and FVIII-LP models showing a pruned Cα root mean square deviation of 0.935 Å (across 1113 atom pairs), confirming that the global observed tilted docking geometry across 2 distinct platforms is an intrinsic feature of the FVIII–membrane interaction. A red dotted box highlights the C2 domain where significant conformational divergence is observed. Top, right panel: A 90° clockwise rotation of the C2 domain from the overlaid models. Bottom, right panel: An additional 45° clockwise turn focusing on the apex of the 2202 loop, illustrating a Cα displacement of up to 8.0 Å at P2205, while residues Met2199 and Phe2200 remain membrane anchored.

**FIGURE 3:**
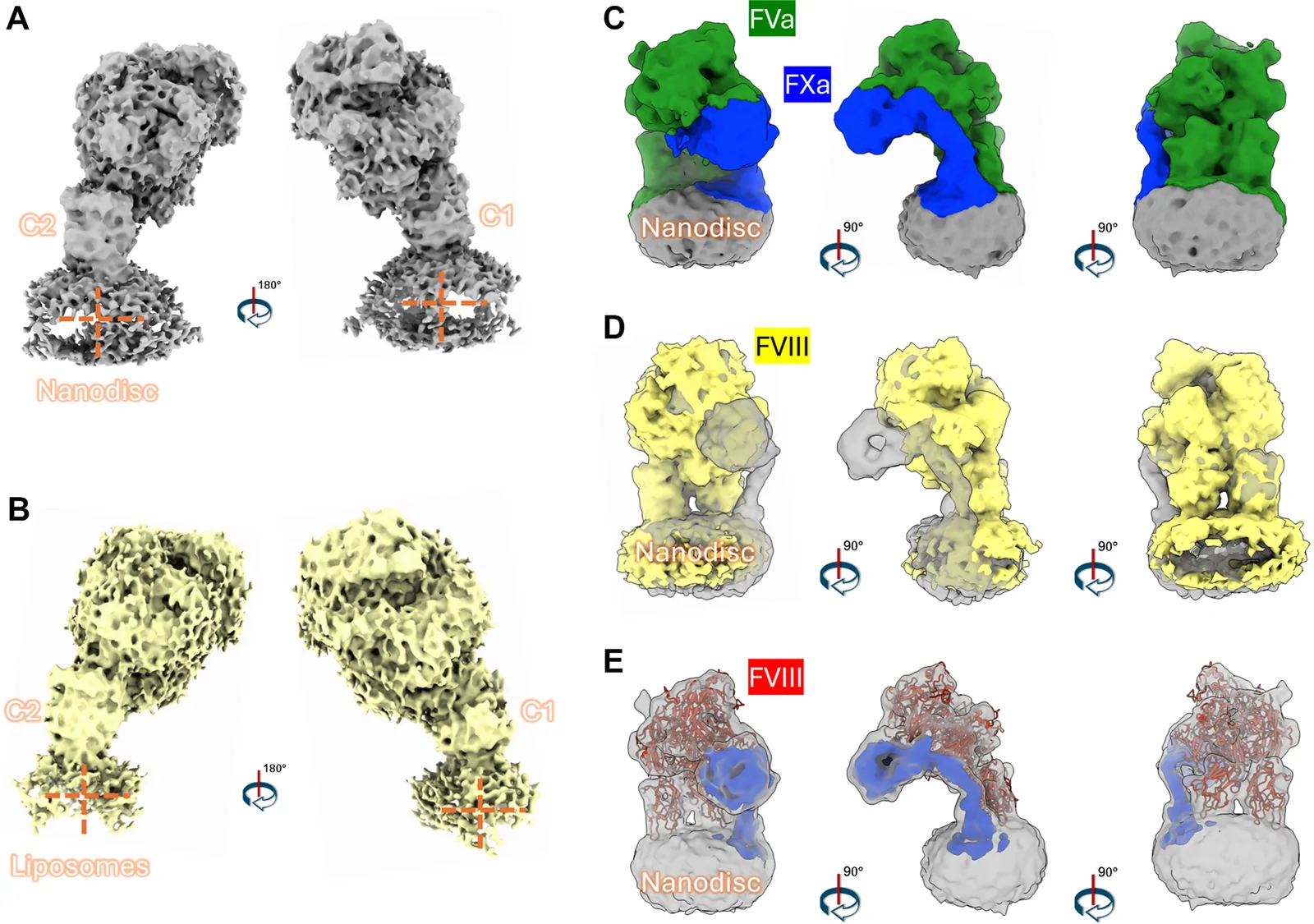
Factor (F)VIII tilted anchoring to the membrane and structural homology to the prothrombinase complex. (A) FVIII–nanodisc (ND) consensus map (EMD-76479) visualized to illustrate the vertical orientation relative to the membrane. An orange crosshair defines the horizontal and vertical membrane axes to highlight the definitive tilted docking angle. The right panel shows a 180° rotation of the map, demonstrating the spatial relationship between the C1 and C2 domains. (B) same as (A) for FVIII–liposome (LP; EMD-76495), demonstrating that the observed tilted geometry is conserved across distinct membrane scaffolds. (C) Cryoelectron microscopy (cryo-EM) map of prothrombinase complex (EMD-42405) showing FVa (green):FXa (blue) (The map has been modified to remove the substrate, prothrombin, density for clarity). Panels show a front view followed by sequential 90° clockwise rotations to visualize the spatial arrangement of the FVa and FXa components. (D) The prothrombinase map (same as in C) rendered transparent gray with the FVIII-ND consensus map (EMD-76479, yellow) rigid-body fitted into it. The fit demonstrates the striking structural convergence and perfect alignment of the FVIII scaffold with the FVa component of the prothrombinase complex. (E) Prothrombinase map (same as in D) map rendered transparent gray with the refined FVIII atomic model (PDB 12JP) docked into the FVa-equivalent density. The corresponding FXa density is highlighted in blue, illustrating how the asymmetric cradle of FVIII serves as a definitive template for modeling the assembly of the intrinsic tenase complex.

**TABLE:** Structural parameters for the cryoelectron microscopy structures of factor (F)VIII on lipid surfaces.

Parameter	FVIII on nanodiscs (FVIII-ND)	FVIII on liposomes (FVIII-LP)
Accession codes		
PDB	PDB 12JP	PDB 12JV
EMDB	EMD-76487	EMD-76497
Data collection and processing		
Nominal magnification	150 000 x	
Voltage (kV)	200	
Electron exposure (e^−^/Å^2^)	55	
Defocus range (μ m)	−1 to −2.5	
Pixel size (Å)	0.928	
No. of images	4185	
Final particle images	58 291	49 208
Symmetry imposed	C1	
Resolution (masked, FSC threshold 0.143) (Å)	3.46	3.56
Model composition		
Refinement program	Phenix (real space)	
No. of protein atoms (non-H)	10 227	10 100
Protein residues	1263	1247
Ligand molecules		
NAG	3	3
Ca	1	1
Cu	2	2
Root mean square deviation		
Bond lengths (Å)	0.003 (0)	0.003 (0)
Bond angles (°)	0.657 (0)	0.634 (0)
Validation		
All-atom clash score	11.04	13.76
Poor rotamers (%)	0.0	0.0
Ramachandran disallowed (%)	0.0	0.0
Ramachandran allowed (%)	6.78	8.03
Ramachandran favored (%)	93.22	91.97
MolProbity score	2.0	2.13

EMDB, Electron Microscopy Data Bank; FSC, Fourier Shell correlation; PDB, Protein Data Bank.
